# Newborn Screening for Biotinidase Deficiency. The Experience of a Regional Center in Italy

**DOI:** 10.3389/fped.2021.661416

**Published:** 2021-05-31

**Authors:** Alice Maguolo, Giulia Rodella, Alice Dianin, Irene Monge, Martina Messina, Erika Rigotti, Francesca Pellegrini, Grazia Molinaro, Fiorenzo Lupi, Andrea Pasini, Natascia Campostrini, Florina Ion Popa, Francesca Teofoli, Monica Vincenzi, Marta Camilot, Giorgio Piacentini, Andrea Bordugo

**Affiliations:** ^1^Department of Mother and Child, University of Verona, Verona, Italy; ^2^Inherited Metabolic Diseases Unit and Regional Centre for Newborn Screening, Diagnosis and Treatment of Inherited Metabolic Diseases and Congenital Endocrine Diseases, Azienda Ospedaliera Universitaria Integrata, Verona, Italy; ^3^Pediatric Clinic Azienda Ospedaliera Universitaria Integrata (AOUI) of Verona, Verona, Italy; ^4^Neonatal Intensive Care Unit, Azienda Sanitaria Alto Adige, Bolzano, Italy; ^5^Department of Pediatrics, The Regional Center for Neonatal Screening, Diagnosis and Treatment of Inherited Congenital Metabolic and Endocrinological Diseases, AOUI, Verona, Italy

**Keywords:** biotinidase enzymatic activity, genotype-phenotype correlation analysis, biotinidase deficiency incidence, biotinidase deficiency, newborn screening, biotinidase deficiency disorder gene

## Abstract

**Introduction:** Biotinidase deficiency (BD) is an autosomal recessive disease causing a defect in the biotin-releasing enzyme. Newborn screening (NBS) allows early diagnosis and treatment, ensuring excellent prognosis. The aim of this study was to describe our experience in the diagnosis, treatment, and follow-up showing key strategies and unsolved questions of the management of BD patients.

**Methods:** We analyzed data of patients identified by the Regional Centre for Newborn Screening of Verona and followed by the Inherited Metabolic Disease Unit of Verona and Neonatal Intensive Care Unit of Bolzano, Italy, from 2014 to 2020.

**Results:** Thirty-seven patients were diagnosed by NBS (five profound and 32 partial BD), with a total incidence of 1:5,996. All were started on biotin at diagnosis and presented no symptoms at follow-up. Analysis of parents and siblings led to identification of five asymptomatic patients with partial BD: one asymptomatic parent and four young siblings. Genetic analysis of the *BTD* gene identified 17 different genotypes and one mutation not previously known.

**Discussion:** Our data confirm that NBS introduction had a dramatic impact on BD diagnosis, and the incidence has increased significantly compared to other areas. Partial defects are more common than profound and have a distinctive genotype. Partial BD treatment is still controversial even at what dose of biotin and for how long. At the end, BD treatment is very easy and inexpensive and prevents severe neurological damage. Sharing experiences is essential to achieving guidelines for treatment and follow-up and a better genotype–phenotype correlation.

## Background

Biotinidase deficiency (BD, OMIM: 253260) is an autosomal recessive disease caused by an altered activity of the enzyme biotinidase. Biotinidase releases biotin from biocytin or small biotinylated peptides downstream the proteolytic turnover of holocarboxylases and other biotinylated proteins. In particular, biotin acts as cofactor for four carboxylation enzymes in the body: 3-methylcrotonyl-CoA carboxylase (MCC), pyruvate carboxylase (PC), acetyl-CoA carboxylase (ACC), and propionyl-CoA carboxylase (PCC).

Based on the residual serum enzyme activity, the defect is distinguished in profound when the residual enzyme activity is < 10% and partial when enzyme activity is between 10 and 30% of mean serum activity calculated in the general population. The incidence reported in literature is about 1:60,000 (1), even if recent authors reported case studies with a significantly higher incidence (2, 3). The clinical presentation is heterogeneous and varies from neurological manifestations such as hypotonia, developmental delay, ataxia, seizures, sensorineural hearing loss, and visual problems, including optic atrophy, to dermatological manifestations such as alopecia, skin rash, and conjunctivitis (4, 5). Patients may present metabolic complications as well, like lactic acidosis, ketoacidosis, and hyperammonemia, and less frequently organic aciduria (5). Some patients may also present respiratory problems, such as hyperventilation, laryngeal stridor, and apnea (4).

Treatment with biotin may not be able to reverse neurological complications as moderate or severe developmental delay, hearing loss, and optic atrophy, especially if a long period has elapsed between their onset and the initiation of treatment (1, 4). On the contrary, dermatological manifestation usually responds favorably to biotin treatment.

As early treatment with oral biotin prevents the onset of clinical symptoms, BD has been successfully included in newborn screening (NBS) programs since 1984 (Commonwealth of Virginia in the United States), allowing for early diagnosis and treatment and ensuring an excellent prognosis of this inborn error of metabolism (5).

While for profound BD the efficacy of NBS and lifelong treatment is a consolidated practice, for partial BD there is a lack of clear indications on the treatment, dosage, and follow-up, resulting in a great heterogeneity between Centers.

The purpose of our study was to describe our experience in the diagnosis, treatment, and follow-up of BD, trying to show the key strategies and unsolved questions of the management of this disease.

## Methods

We carried out a retrospective analysis of patients affected by BD identified from January 2014 to December 2020 by the Metabolic Hereditary Diseases Center of Verona.

NBS for BD have been performed since 1986 at the Regional Center for Neonatal Screening, Diagnosis and Treatment of Inherited Congenital Metabolic and Endocrinological Diseases of Verona covering North East of Italy while, from 2014, the Inherited Metabolic Diseases Unit is actively involved in treatment and follow-up of children diagnosed with total and partial biotinidase deficiency. The newborn dried blood spots (DBS) were collected at 36–72 h of life. If the first DBS value was below cutoff, a second sample was requested and possibly a third one. Until December 2014, the enzyme activity on DBS was analyzed with a non-quantitative colorimetric method and afterward was semi-quantitatively measured with the GSP Neonatal Biotinidase kit (Perkin Elmer, Wallac Oy), an assay combining an enzyme reaction with a solid phase time-resolved immunofluorescence assay.

The diagnostic confirmation was performed by the analysis of serum activity (6) and by molecular analysis of the *BTD* gene in all probands and parents.

Patient serum has been separated from whole blood within 2 h and sent to the laboratory in dry ice, together with the parents' samples and a non-family-related serum specimen collected and sent for transport condition control purposes. Upon lab arrival, all of them were stored at −80°C for a maximum of 2 weeks. Serum biotinidase activity has been measured by a colorimetric assay using the artificial substrate N(+)-biotinyl-4-aminobenzoic acid (B-PABA): the enzyme cleaves the amide bond of B-PABA, freeing biotin and p-aminobenzoic acid (PABA). PABA is then converted to a purple compound, easily spectrophotometrically quantitated. No color develops when biotinidase is either missing or completely inactive.

Quality check of the batch has been performed by including a serum sample of an individual with normal biotinidase activity. Such a sample has been stored at −70°C in aliquots and thawed just once. Furthermore, a positive control has been prepared and included in each batch as well, by heating a serum sample for 1 h at 60°C.

Genomic DNA was extracted from patients' peripheral venous blood on EDTA by means of the QIAmp DNA Blood Mini kit (QIAGEN S.p.A, Milan, Italy), following the manufacturer's instructions. All exons and part of the flanking intron regions of the *BTD* gene (NM_000060.4) were amplified by polymerase chain reactions and sequenced for molecular analysis (primers available upon request). For each variant identified, databases available online have been used to verify if mutations were already reported in literature, such as Pubmed, dbSNP, ClinVar, and Human Genetic Variation Database (HGVD). If not previously reported, *in silico* prediction analyses were performed for missense variants using PolyPhen-2 (http://genetics.bwh.harvard.edu/pph2) and Mutation Taster (http://www.mutationtaster.org/) tools.

Therapy with biotin was started at a dosage of 10 mg/day for profound or 5–10 mg/day for partial BD in all patients with the suggestion to eliminate raw eggs, containing avidin from the diet, a protein interacting with biotin and decreasing its bioavailability (4).

The patients underwent the following biochemical and clinical follow-up: blood count with formula, biochemical profile with lactate, ammonia, blood gas analysis, audiometry, eye examination, and pediatric evaluation to assess growth and neurological development and look for skin problems. All were carried out every year for profound BD and every 2 years for partial BD (4). Furthermore, patients with profound BD underwent urinary organic acids evaluation every year to monitor efficacy and compliance to therapy (1, 4).

## Results

### Patients

Among 293,784 newborns screened by the Regional Screening Centre of Verona since 2014 until the end of 2020, 287 were recalled to repeat DBS in cases of suspected BD and 49 were diagnosed with BD, with a total incidence of 1:5,996 newborns. In particular, an incidence of 1:58,757 for profound BD and 1:6,677 for partial BD were found. A total of five (10.2%) patients with profound BD was detected. Thirty-four patients were followed up in Verona (four patients with profound and 30 with partial BD) and three patients (one profound and two partial BD) in Bolzano, and 12 remaining patients were followed up in another center. Eighteen patients were male (48.6%), and 19 patients were female (51.4%). All of them, identified by NBS, started biotin supplementation at diagnosis at the dosage of 10 mg/day for profound BD and 5–10 mg/day for partial deficiencies.

None of the patients, profound BD included, presented signs or symptoms related to BD at diagnosis or at clinical follow-up (Table 1). Psychomotor development and auditory and visual functions were normal, and no relevant dermatological problems attributable to the deficiency were detected. Only patient 3 with partial BD presented language delay at 2.5 years, despite proper therapy, not related to BD. The mean follow-up period was 43.3 months +/– 23.0 standard deviation.

**Table 1:** Diagnosis, clinical and genetic characterization of patients affected by biotinidase deficiency.

**Id**	**Duration of follow up (months)**	**Age (years) at follow-up**	**M/F**	**Diagnosis**	**Biotinidase enzimatic activity on DBS (U/dL)**	**Biotinidase enzymatic activity on serum (nmol/pABA/min/mL)**	**Allele 1**	**Molecular consequence**	**Reported citation or pathogenic classification**	**Allele 2**	**Molecular consequence**	**Reported citation or pathogenic classification**	**Type of deficit**	**Free biotin treatment**	**Audiometric evaluation**	**Ophthalmologic evaluation**	**Neurological development assessment**	**Dermatological evaluation**	**Degree of kinship**
pt 1	73	6.1	F	NBS	52	2.7	c.98_104 delGCG GCTGin sTCC p.Cys33Phefs	Frameshift	Pomponio et al. (7)	c.1330G>C p.Asp444His	Missense	Swango et al. (8)	Partial	5 mg × 2/die	Normal	Normal	Normal	Atopic dermatitis	N/A
pt 2	26	2.1	M	NBS	74	2.7	c.1613G>T p.Arg538Leu	Missense	Uncertain clinical significance	c.1330G>C p.Asp444His	Missense	Swango et al. (8)	Partial	5 mg × 2/die	Normal	Normal	Normal	Normal	N/A
pt 3	35	2.9	M	NBS	55.7	2.7	c.1368A>C p.Gln456His	Missense	Norrgard et al. (9)	c.1330G>C p.Asp444His	Missense	Swango et al. (8)	Partial	5 mg × 2/die	N/A	N/A	Language regression	Normal	Sibling of pt 38-39
pt 4	33	2.7	F	NBS	44	2.9	c.454A>C p.Thr152Pro	Missense	Milánkovics et al. (10)	c.1330G>C p.Asp444His	Missense	Swango et al. (8)	Partial	5 mg × 2/die	Normal	Normal	Normal	Normal	N/A
pt 5	23	1.8	M	NBS	66	2.9	c.98_104 delGCGGC TGinsTCC p.Cys33Phefs	Frameshift	Pomponio et al. (7)	c.1330G>C p.Asp444His	Missense	Swango et al. (8)	Partial	5 mg × 2/die	N/A	N/A	Normal	Normal	N/A
pt 6	70	5.8	F	NBS	56	1.3	c.[470G>A; 1330G>C] p.(Arg157His; Asp444His)	Missense	Norrgard et al. (11)	c.1330G>C p.Asp444His	Missense	Swango et al. (8)	Partial	5 mg × 2/die	Normal	Normal	Normal	Normal	N/A
pt 7	53	4.4	F	NBS	39	2.2	c.1368A>C p.Gln456His	Missense	Norrgard et al. (9)	c.1330G>C p.Asp444His	Missense	Swango et al. (8)	Partial	5 mg × 2/die	Transmissive hypoacusia	Hyper metropia	Normal	Atopic dermatitis	N/A
pt 8	27	2.2	F	NBS	51	2.3	c.341G>T p.Gly114Val	Missense	Wolf et al. (12)	c.1330G>C p.Asp444His	Missense	Swango et al. (8)	Partial	5 mg × 2/die	Normal	Normal	Normal	Normal	N/A
pt 9	38	3.1	M	NBS	44.5	2.1	c.[470G>A; 1330G>C] p.(Arg157His; Asp444His)	Missense	Norrgard et al. (11)	c.1330G>C p.Asp444His	Missense	Swango et al. (8)	Partial	5 mg × 2/die	Normal	Normal	Normal	Eczema	N/A
pt 10	65	5.4	M	NBS	42	2.4	c.1368A>C p.Gln456His	Missense	Norrgard et al. (9)	c.1330G>C p.Asp444His	Missense	Swango et al. (8)	Partial	5 mg × 1/die	Left trans missive deafness	Normal	Normal	Normal	N/A
pt 11	64	5.3	M	NBS	47.5	2.9	c.[511G>A; 1330G>C] p.(Ala171Thr; Asp444His)	Missense	Norrgard et al. (11)	c.1330G>C p.Asp444His	Missense	Swango et al. (8)	Partial	5 mg × 1 /die	Normal	Normal	Normal	Normal	N/A
pt 12	17	1.4	M	NBS	42	2.5	c.98_104del GCGGCT GinsTCC p.Cys33Phefs	Frameshift	Pomponio et al. (7)	c.1330G>C p.Asp444His	Missense	Swango et al. (8)	Partial	5 mg × 2/die	Normal	N/A	Normal	Normal	N/A
pt 13	41	3.4	M	NBS	52.3	2.2	c.[511G>A; 1330G>C] p.(Ala171Thr; Asp444His)	Missense	Norrgard et al. (11)	c.1330G>C p.Asp444His	Missense	Swango et al. (8)	Partial	5 mg × 2/die	Normal	Normal	Normal	Normal	N/A
pt 14	67	5.5	M	NBS	53.5	2.7	c.218C>T p.Pro73Leu	Missense	Canda et al. (13)	c.1330G>C p.Asp444His	Missense	Swango et al. (8)	Partial	5 mg × 2/die	Normal	Exophoria	Normal	Normal	N/A
pt 15	63	5.2	F	NBS	46.5	2.6	c.1368A>C p.Gln456His	Missense	Norrgard et al. (9)	c.1330G>C p.Asp444His	Missense	Swango et al. (8)	Partial	5 mg × 1/die	Normal	Normal	Normal	Normal	N/A
pt 16	66	5.5	F	NBS	45	1.7	c.1595C>T p.Thr532Met	Missense	Seker Yilmaz et al. (14)	c.1330G>C p.Asp444His	Missense	Swango et al. (8)	Partial	5 mg × 2/die	Normal	N/A	Normal	Normal	N/A
pt 17	33	2.7	F	NBS	55	2.8	c.[511G>A; 1330G>C] p.(Ala171Thr; Asp444His)	Missense	Norrgard et al. (11)	c.1330G>C p.Asp444His	Missense	Swango et al. (8)	Partial	5 mg × 2/die	Normal	N/A	Normal	Mild dermatitis	Daughter of pt 40
pt 18	68	5.6	M	NBS	43	2.9	c.98_104del GCGGCTG insTCC p.Cys33Phefs	Frameshift	Pomponio et al. (7)	c.1330G>C p.Asp444His	Missense	Swango et al. (8)	Partial	5 mg × 2/die	Normal	Normal	Normal	Normal	N/A
pt 19	16	1.3	F	NBS	62	2.5	c.278A>Gp. Tyr93Cys	Missense	Wolf et al. (15)	c.1330G>C p.Asp444His	Missense	Swango et al. (8)	Partial	5 mg × 2/die	N/A	N/A	Normal	Normal	N/A
pt 20	21	1.7	M	NBS	58	2.8	c.98_104del GCGGCTG insTCC p.Cys33Phefs	Frameshift	Pomponio et al. (7)	c.1330G>C p.Asp444His	Missense	Swango et al. (8)	Partial	5 mg × 1/die	Normal	N/A	Normal	Normal	Sibling of pt 32
pt 21	20	1.6	M	NBS	42.5	2.7	c.1595C>T p.Thr532Met	Missense	Seker Yilmaz et al. (14)	c.1330G>C p.Asp444His	Missense	Swango et al. (8)	Partial	5 mg × 2/die	Normal	normal	Normal	Normal	N/A
pt 22	73	6.1	M	NBS	N/A	1.3	c.[511G>A; 1330G>C] p.(Ala171Thr; Asp444His)	Missense	Norrgard et al. (11)	c.1330G>C p.Asp444His	Missense	Swango et al. (8)	Partial	5 mg × 2/die	Normal	N/A	Normal	Normal	Sibling of pt 41
pt 23	56	4.7	F	NBS	36.5	2.2	c.1368A>C p.Gln456His	Missense	Norrgard et al. (9)	c.1330G>C p.Asp444His	Missense	Swango et al. (8)	Partial	5 mg × 2/die	Normal	Normal	Normal	Normal	N/A
pt 24	20	1.6	M	NBS	53.3	1.7	c.98_104del GCGGCTG insTCC p.Cys33Phefs	Frameshift	Pomponio et al. (7)	c.1330G>C p.Asp444His	Missense	Swango et al. (8)	Partial	5 mg × 2/die	N/A	N/A	Normal	Normal	N/A
pt 25	19	1.6	M	NBS	53	2.7	c.631C>T p.Arg211Cys	Missense	Norrgard et al. (11)	c.1330G>C p.Asp444His	Missense	Swango et al. (8)	Partial	5 mg × 2/die	N/A	Normal	Normal	Normal	Sibling of pt 42
pt 26	66	5.5	F	NBS	36.5	2.3	c.334G>A p.Glu112Lys	Missense	Laszlo et al. (16)	c.1330G>C p.Asp444His	Missense	Swango et al. (8)	Partial	5 mg × 2/die	Normal	Normal	Normal	Normal	N/A
pt 27	31	2.6	M	NBS	44.5	2.5	c.98_104del GCGGCTG insTCC p.Cys33Phefs	Frameshift	Pomponio et al. (7)	c.1330G>C p.Asp444His	Missense	Swango et al. (8)	Partial	5 mg × 2/die	Normal	Normal	Normal	Normal	N/A
pt 28	31	2.5	F	NBS	53.7	2.2	c.98_104del GCGGCTG insTCC p.Cys33Phefs	Frameshift	Pomponio et al. (7)	c.1330G>C p.Asp444His	Missense	Swango et al. (8)	Partial	5 mg × 2/die	Normal	Normal	Normal	Normal	N/A
pt 29	5	0.3	F	NBS	49.5	2.1	c.1368A>C p.Gln456His	Missense	Norrgard et al. (9)	c.1330G>C p.Asp444His	Missense	Swango et al. (8)	Partial	5 mg × 2/die	N/a	N/A	Normal	Normal	N/A
pt 30	4	0.2	F	NBS	60	1.2	c.[511G>A; 1330G>C] p.(Ala171Thr; Asp444His)	Missense	Norrgard et al. (11)	c.1330G>C p.Asp444His	Missense	Swango et al. (8)	Partial	5 mg × 2/die	N/a	N/A	Normal	Normal	N/A
pt 31	90	7.6	F	NBS	N/A	2.4	c.[511G>A; 1330G>C] p.(Ala171Thr; Asp444His)	Missense	Norrgard et al. (11)	c.1330G>C p.Asp444His	Missense	Swango et al. (8)	Partial	5 mg × 2/die	Normal	N/A	Normal	Normal	N/A
pt 32	65	5.4	M	NBS	42.5	2.7	c.98_104del GCGGCTG insTCC p.Cys33Phefs	Frameshift	Pomponio et al. (7)	c.1330G>C p.Asp444His	Missense	Swango et al. (8)	Partial	5 mg × 1/die	Normal	N/A	Normal	Normal	Sibling of pt 20
pt 33	74	6.1	M	NBS	8.5	0.7	c.508G>A p.Val170Met	Missense	Likely pathogenic by prediction tools	c.508G>A p.Val170Met	Missense	Likely pathogenic by prediction tools	Profound	10 mg × 2/die	Normal	Normal	Normal	Normal	N/A
pt 34	43	3.5	F	NBS	15	1.1	c.1489C>T p.Pro497Ser	Missense	Sarafoglou et al. (17)	c.1489C>T p.Pro497Ser	Missense	Swango et al. (8)	Profound	5 mg × 2/die	Normal	Normal	Normal	Normal	N/A
pt 35	45	3.7	F	NBS	9	0.7	c.98_104del GCGGCTG insTCC p.Cys33Phefs	Frameshift	Pomponio et al. (7)	c.98_104del GCGGCTG insTCC p.Cys33Phefs	Frameshift	Pomponio et al. (7)	Profound	5 mg × 2/die	Normal	N/A	Normal	Normal	N/A
pt 36	20	1.6	F	NBS	8	0.5	c.184G>A p.Val62Met	Missense	Muhl et al. (18)	c.[511G>A; 1330G>C] p.(Ala171Thr; Asp444His)	Missense	Swango et al. (8)	Profound	5 mg × 2/die	Normal	N/A	Normal	Normal	N/A
pt 37	71	5.9	F	NBS	7	0.2	c.1612C>T p.Arg538Cys	Missense	Pomponio et al. (7)	c.1612C>T p.Arg538Cys	Missense	Swango et al. (8)	Profound	5 mg × 2/die	Normal	Normal	Normal	Normal	N/A
pt 38	35	13.0	M	Familial screening	N/A	1.7	c.1368A>C p.Gln456His	Missense	Norrgard et al. (9)	c.1330G>C p.Asp444His	Missense	Swango et al. (8)	Partial	5 mg × 2/die	Normal	N/A	Mild language delay and learning disability	Normal	Sibling of pt 3
pt 39	35	8.5	M	Familial screening	N/A	2.9	c.1368A>C p.Gln456His	Missense	Norrgard et al. (9)	c.1330G>C p.Asp444His	Missense	Swango et al. (8)	Partial	5 mg × 2/die	N/A hypoacusia?	N/A	Normal	Normal	Sibling of pt 3
pt 40	32	37.8	F	Familial screening	N/A	2.9	c.[511G>A; 1330G>C] p.(Ala171Thr; Asp444His)	Missense	Norrgard et al. (11)	c.1330G>C p.Asp444His	Missense	Swango et al. (8)	Partial	No treatment	Normal	Normal	Normal	Normal	Mother of pt 17
pt 41	72	11.6	M	Familial screening	N/A	2.1	c.[511G>A; 1330G>C] p.(Ala171Thr; Asp444His)	Missense	Norrgard et al. (11)	c.1330G>C p.Asp444His	Missense	Swango et al. (8)	Partial	5 mg × 2/die	Normal	N/A	Normal	Dermatitis	Sibling of pt 22
pt 42	7	11.1	F	Familial screening	N/A	2.6	c.631C>T p.Arg211Cys	Missense	Norrgard et al. (11)	c.1330G>C p.Asp444His	Missense	Swango et al. (8)	Partial	5 mg × 2/die	N/A	N/A	Normal	Normal	Sibling of pt 25

*In column of DBS is reported the average value of repeated analysis. DBS, dried blood spot; NBS, newborn screening; N/A, not applicable or not available; M, male; F, female. Reference sequences: NM_000060.4; NP_000051.1*.

Five patients with partial BD were identified through family study: one asymptomatic parent of a proband (35 years of age at diagnosis) and four siblings of patients (Table 1). They promptly started therapy with oral biotin at the dosage of 10 mg a day, except for the adult parent that remained asymptomatic throughout her life. Two siblings of patient 3, 10, and 6 years at diagnosis, presented with a history of mild language delay with learning disability and a reported hearing impairment ongoing assessment, respectively.

A third familiar case, sibling of patient 22 identified at 6 years of age, presented at the diagnosis a dermatitis, not previously investigated which improved with moisturizing lotions.

### Molecular Analysis

Genetic analysis of the gene *BTD* was completed in all 42 patients (Table 1).

**Figure 1:**
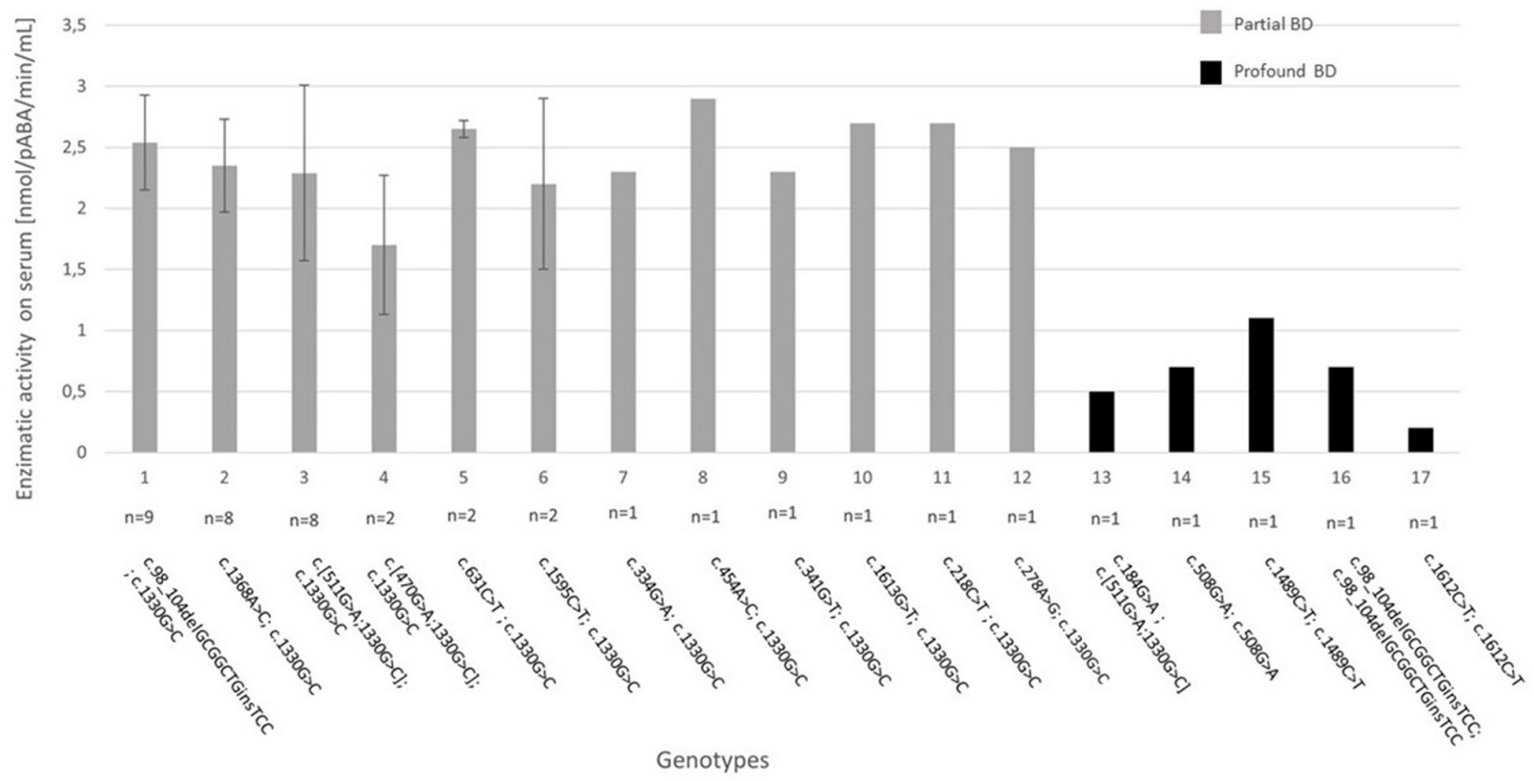
Genotype–biochemical phenotype correlation for the 17 genotypes identified in our study. The enzymatic activity on serum is expressed as absolute value when *n* = 1 or mean +/– standard deviation when *n* > 1. BD, biotinidase deficiency. Reference sequence for variants: NM_000060.4.

We identified 17 different genotypes in the entire group, which are presented in Figure 1. The most frequent genotype was found in nine patients (21.4%) with partial BD and is characterized by the frameshift mutation c.98_104delGCGGCTGinsTCC (p.Cys33Phefs) and the c.1330G>C (p.Asp444His) variant on the *BTD* gene present in compound heterozygosity, both already reported in literature (7, 8). The second two most frequent genotypes, identified in eight patients with partial BD each (19.0%), were characterized by c.1368A>C (p.Gln456His) and c.1330G>C (p.Asp444His) on the *BTD* gene present in compound heterozygosity, both already reported in literature (8, 9); while the second one was characterized by the already known mutation c.511G>A (p.Ala171Thr) (11) and the c.1330G>C (p.Asp444His) variant in cis and c.1330G>C (p.Asp444His) present in the other allele.

The known mutation c.1330G>C (p.Asp444His) has been identified in all patients with partial BD, with a pathogenic variant in compound heterozygosity. This is a mutation with high prevalence in the European population (about 4%) (19), which produces an enzyme with about 50% of residual activity (20).

Two patients affected by profound BD are homozygous for common mutations c.1612C>T (p.Arg538Cys) and c.98_104delGCGGCTinsTCC (p. Cys33Phefs), known in literature to be found in about 30 and 50% of patients with profound deficiency, respectively (7). Pt 34 is homozygous for the mutation c.1489C> T (p.Pro497Ser), already reported in literature associated with profound BD (17). In this case, genetic analysis helped us to diagnose this patient as profound deficiency despite a borderline enzymatic activity of 1.1 nmol/pABA/min/mL at the diagnosis. Pt 36 carries the known c.184G>A (p.Val62Met) variant (18) in compound heterozygosity with the c.511G>A (p.Ala171Thr) and c.1330G>C (p.Asp444His) variants in cis, and patient 33 is homozygous for the unknown substitution c.508G>A (p.Val170Met). Both patients 33 and 34 have certain degrees of consanguinity of the parents in their medical history.

Hence, a mutation never previously reported in literature has been identified in our study: c.508G>A (p.Val170Met) in homozygosity in patient 33 with profound BD (enzymatic activity 0.7 nmol/pABA/min/mL) predicted likely pathogenic by prediction tools. In contrast, patient 2 presented the c.1613G>T (p.Arg538Leu) missense variant that has been reported in dbSNP rs397514429 with uncertain clinical significance but in our study is clearly associated with partial BD (enzymatic activity 2.7 nmol/pABA/min/mL) when in compound heterozygosity with the common variant c.1330G>C (p.Asp444His).

## Discussion

From our data, a remarkable overall incidence of 1:5,996 newborns with BD, combining partial and total deficiencies, emerged. Our incidence is significantly higher than previously reported in literature (5), even if recently some authors reported case studies identified by NBS with comparable incidences in different countries (2, 3, 13, 21).

All patients identified by NBS, thanks to the timely initiation of therapy and follow-up, did not present any clinical signs and symptoms related to the spectrum of BD. Although our data come from a fairly short follow-up, a recent study confirms this finding by reporting adolescents and adults, ages 16–32 years old, with profound biotinidase deficiency ascertained by NBS with excellent outcomes (22).

While the fundamental usefulness of NBS in ensuring early diagnosis and good prognosis for profound BD is unquestionable, the management of patients with partial BD diagnosed thanks to NSB is still a matter of discussion. Although they may show milder symptoms, most patients with partial BD remain asymptomatic throughout life (23). Additionally, milder symptoms are usually characterized mainly by cutaneous signs such as rash and alopecia, which are usually reversible with the initiation of therapy (24).

However, there have been reported in literature patients with partial BD not taking biotin with more severe clinical pictures, identified either by screening or clinically (20). Wolf and colleagues in 2015 reported several patients with partial BD with a clinical onset characterized by a severe neurological symptomatology with seizures, psychomotor development delayed, hearing loss, and ataxia (20).

Therefore, patients with partial BD could develop even severe clinical symptoms, if not treated, especially during stressful events, like severe infections (13, 20). It is precisely for this reason, and given the absence of side effects associated with biotin supplementation at commonly used doses, that there is currently consensus on starting oral biotin supplementation even in patients with partial BD. Nonetheless, there are still no precise guidelines on the proper dosage of oral biotin supplementation, the duration of the therapy, and the need and timing of clinical and biochemical follow-up.

In some case series, patients with profound BD were treated with up to 15–20 mg of biotin per day, with the indication to continue lifelong. Although there are no clear indications, particularly for partial deficits, it seems reasonable to start with a dosage of 10 mg per day in a single administration or divided in two administration for profound BD and with a dosage ranging between 1 and 10 mg per day for partial deficiencies, possibly to increase according to the appearance of symptomatology (1, 22).

All our partial BD patients identified by NBS were persistently asymptomatic during follow-up, with normal psychomotor development, except for patient 3, belonging to a familiar case discussed thoroughly later. Auditory and visual problems, likewise dermatological problems, revealed at the follow-up evaluations of our NBS patients, were not attributable to BD such as transmissive hypoacusia, orthoptics problems, atopic dermatitis, and transient eczema, as reported in Table 1.

Instead, partial BD identified by familiar screening at diagnosis presented some phenotypes, but it is difficult to assert if it was caused by BD. The two siblings identified at the age of 10 and 6 years, after the diagnosis of patient 3 by NBS, have presented a history of mild language delay with learning disability and hypoacusia, ongoing assessments. They have started biotin therapy after NBS result of patient 3, without any symptomatology improvement. Also, patient 3 developed delayed speech at the age of 2.5 years, similarly to one brother, despite good compliance in therapy with biotin from birth. It is therefore plausible that the familial phenotype was not caused by BD, and further diagnostic investigations are required to define the etiology.

Four patients among those detected in the family analysis were not identified by NBS, although they were born after its introduction in our region. This is likely due to the old non-quantitative colorimetric method which has been discontinued since December 2014 in favor of the enzymatic and immunofluorescence reaction. In fact, comparison studies suggested that the fluorescence method was slightly more specific and sensitive than the colorimetric assay (25).

It is therefore important to underline the relevance that the clinical diagnosis of BD still has nowadays. In fact, BD must be recognized and considered in the differential diagnosis in case of symptomatic patients with late onset who not only may not have been identified at screening, but who may belong to the pre-NBS era, since in most cases these patients respond to biotin treatment (4).

Our genetic data confirmed that partial BD are more frequent than profound (88.1%) and are characterized by a distinctive genotype with a high prevalence of the c.1330G>C (p.Asp444His) variant (8, 19). Essentially, all individuals with partial BD have the mutation c.1330G>C (p.Asp444His) in one or both alleles of the *BTD* gene in combination with a mutation associated with profound BD in heterozygosity, and this is in agreement with other studies already reported in the literature (10, 14, 16, 21).

The high frequency of c.1330G>C (p.Asp444His) is characteristic of European populations, with existing geographical differences in frequencies observed in other countries (19).

Genetic analysis is useful for the diagnostic confirmation of these defects; particularly, it can be decisive in the case of borderline enzymatic activity (4). Our patient 34 with a borderline serum enzymatic activity of 1.1 nmol/pABA/min/mL at diagnosis has eventually been defined as profound BD, thanks to the finding of a genotype characterized by the c.1489C> T (p.Pro497Ser) homozygous mutation, already associated in literature with profound BD (17). This genotype–phenotype correlation was further confirmed by a repeated enzymatic analysis on serum, which resulted < 10% of the mean calculated in the general population.

Similarly, genetic analysis may be useful if the serum enzymatic activity is approximately 30% of normal activity to discriminate between heterozygous and partial BD and decide accordingly whether to start treatment (19, 21).

Although genetic analysis confirmed and supported the diagnosis, particularly in cases of borderline enzymatic activity, further investigations are needed to elucidate a clear genotype–phenotype correlation for BD. In fact, in literature the association between the *BTD* genotype and biotinidase activity is not always consistent (2, 3, 19) and decisions regarding treatment should be based primarily on enzymatic activity (4). This disagreement may be due to factors that influence the biotinidase activity assay, as well as to genetic factors that currently remain unknown such as the presence of variants in non-coding regions of the *BTD* gene (19). Anyway, in our case study, genotype–biochemical phenotype association was quite reliable for patients identified by NBS. In fact, the three most numerically represented genotypes all have overlap serum enzymatic activity values, as it is possible to see from the small standard deviations reported in Figure 1.

From our case series, a high molecular heterogeneity emerged: on 42 patients, genetic analysis of the *BTD* gene identified 17 different genotypes and one mutation not previously reported in the literature and predicted as likely pathogenic by prediction tools.

## Conclusion

BD can be properly diagnosed and treated through NBS Programs. Treatment is inexpensive and easy to go. On the other hand, neurological consequences of a missed diagnosis may be dramatic for children and families.

This should prompt all Countries all around the world to implement in their screening policies BD identification through enzyme activity on DBS. However, it is of paramount importance to consider BD in the differential diagnosis of patients with late onset, particularly with neurological symptoms, as early treatment with biotin can reverse the clinical picture.

After the inclusion of BD in the NBS program, the number of diagnosed patients has increased significantly, especially for partial BD. Currently, there are no European or international management guidelines so there is great heterogeneity in the dose used for treatment and in the follow-up for these patients. Furthermore, given the relatively recent introduction of NBS, there are still few studies with long follow-ups that allow us to understand the clinical course of these patients, especially for what concern adult ages.

Although the measurement of enzymatic activity remains the major tool for the diagnosis of BD, our experience confirms that genetic analysis is useful for genotype–phenotype correlation study and for the diagnostic confirmation, particularly in cases of borderline enzymatic activity. Nonetheless, further studies are needed to improve our knowledge about genotype–phenotype correlations for BD. On the other end, only by bringing together the experience of multiple centers and sharing databases by combining clinical information, enzymatic activity, and DNA sequence will it be possible to develop guidelines and improving the management of BD patients.

## Data Availability Statement

The original contributions presented in the study are included in the article/supplementary materials, further inquiries can be directed to the corresponding author/s.

## Ethics Statement

Ethical review and approval was not required for the study on human participants in accordance with the local legislation and institutional requirements. The patients/participants [legal guardian/next of kin] provided written informed consent to participate in this study.

## Author Contributions

All authors are responsible for reported research. All authors have participated in the concept and design, analysis and interpretation of data, and drafting or revising of the manuscript, and they have approved the manuscript as submitted.

## Conflict of Interest

The authors declare that the research was conducted in the absence of any commercial or financial relationships that could be construed as a potential conflict of interest. The handling Editor declared a past co-authorship with one of the authors AB.

## Abbreviations

BD: Biotinidase deficiency
NBS: Newborn screening
DBS: Dried blood spot.
